# Eimeripain, a Cathepsin B-Like Cysteine Protease, Expressed throughout Sporulation of the Apicomplexan Parasite *Eimeria tenella*

**DOI:** 10.1371/journal.pone.0031914

**Published:** 2012-03-22

**Authors:** Anaïs Rieux, Simon Gras, Fabien Lecaille, Alisson Niepceron, Marilyn Katrib, Nicholas C. Smith, Gilles Lalmanach, Fabien Brossier

**Affiliations:** 1 INRA, UMR1282, Equipe Pathogenèse des Coccidioses, Infectiologie et Santé Publique, Nouzilly, France; 2 Université François Rabelais de Tours, UMR1282, Infectiologie et Santé Publique, Tours, France; 3 INSERM U618, Protéases et Vectorisation Pulmonaires, Université François Rabelais, Tours, France; 4 Institute for the Biotechnology of Infectious Diseases, University of Technology, Sydney, Australia; 5 Queensland Tropical Health Alliance, Faculty of Medicine, Health and Molecular Sciences, James Cook University, Cairns, Australia; Hospital for Sick Children, Canada

## Abstract

The invasion and replication of *Eimeria tenella* in the chicken intestine is responsible for avian coccidiosis, a disease that has major economic impacts on poultry industries worldwide. *E. tenella* is transmitted to naïve animals via shed unsporulated oocysts that need contact with air and humidity to form the infectious sporulated oocysts, which contain the first invasive form of the parasite, the sporozoite. Cysteine proteases (CPs) are major virulence factors expressed by protozoa. In this study, we show that *E. tenella* expresses five transcriptionally regulated genes encoding one cathepsin L, one cathepsin B and three cathepsin Cs. Biot-LC-LVG-CHN_2_, a cystatin derived probe, tagged eight polypeptides in unsporulated oocysts but only one in sporulated oocysts. CP-dependant activities were found against the fluorescent substrates, Z-FR-AMC and Z-LR-AMC, throughout the sporulation process. These activities corresponded to a cathepsin B-like enzyme since they were inhibited by CA-074, a specific cathepsin B inhibitor. A 3D model of the catalytic domain of the cathepsin B-like protease, based on its sequence homology with human cathepsin B, further confirmed its classification as a papain-like protease with similar characteristics to toxopain-1 from the related apicomplexan parasite, *Toxoplasma gondii*; we have, therefore, named the *E. tenella* cathepsin B, eimeripain. Following stable transfection of *E. tenella* sporozoites with a plasmid allowing the expression of eimeripain fused to the fluorescent protein mCherry, we demonstrated that eimeripain is detected throughout sporulation and has a punctate distribution in the bodies of extra- and intracellular parasites. Furthermore, CA-074 Me, the membrane-permeable derivative of CA-074, impairs invasion of epithelial MDBK cells by *E. tenella* sporozoites. This study represents the first characterization of CPs expressed by a parasite from the *Eimeria* genus. Moreover, it emphasizes the role of CPs in transmission and dissemination of exogenous stages of apicomplexan parasites.

## Introduction

*Eimeria spp.* are enteropathogens that infect a variety of mammals and birds. Avian coccidiosis is caused by the infection of chicken with extremely well adapted *Eimeria spp.* These infectious diseases lead to major economic losses worldwide in poultry industries. Anticoccidial drugs are used extensively to control the disease but the increase of resistant parasite populations underlines the need to find alternative targets and drugs.

The genus, *Eimeria*, belongs to the phylum Apicomplexa, a group of medically and economically important parasites including *Plasmodium spp.* (agents of malaria) and *Toxoplasma gondii* (the agent of toxoplasmosis). Among the seven species of *Eimeria* commonly detected in infected chickens, *Eimeria tenella* is one of the most virulent and the only one for which the genome has been sequenced (http://www.genedb.org/Homepage/Etenella) [1]. As for other Coccidia, the complex life cycle of *E. tenella* is divided into an intestinal and an environmental stage. The intestinal stage involves the invasion and replication of parasites within epithelial cells of the chicken intestine, followed by production of male and female gametes, fertilisation and formation of unsporulated oocysts, which are released into the environment. The environmental stage involves maturation, also called sporulation, of unsporulated oocysts into infectious sporulated oocysts. Each sporulated oocyst contains eight haploid sporozoites, the first invasive form of the parasite.

Cysteine cathepsins related to papain-like enzymes, (clan CA, family C1) are major virulence factors expressed by parasites, including apicomplexan parasites ([2] and see the MEROPS database - http://merops.sanger.ac.uk/cgi-bin/clan_index?type=P). Thus, several studies have revealed the crucial roles of cysteine cathepsins (including cathepsin B- and L-like proteases) in cell invasion [3], [4], [5], nutrient acquisition [6], [7], [8], replication and egress [9], [10] by apicomplexan parasites. Cysteine cathepsins may also play a role in the biology of sexual stages and/or the development of oocyst [11] since, for example, a *falcipain-1* knock-out strain of *Plasmodium falciparum* is not affected in its capacity to replicate within the erythrocyte but is impaired in its capacity to produce oocysts [12]. It is also known that, in oocysts of *Eimeria*, an orthologue of a *T. gondii* cathepsin B, toxopain-1 [13], as well as an aminopeptidase [14], [15], and a serine protease from the rhomboid family [16] are all expressed at various times during sporulation of oocysts, though a definitive role for proteases in this process has not been demonstrated.

In this study, we describe the biochemical and molecular characterization of CPs expressed in the environmental oocyst stages of *E. tenella* as it undergoes sporulation and produces the invasive sporozoite stage. We have specifically identified a cathepsin B-like protease and investigated its expression profile, biochemical properties, localization and role in cell invasion.

## Results

### Identification and expression profile of CP genes expressed by *E. tenella*

Five putative CP genes including one *cathepsin B* (this work, Accession no JN641867, on Supercontig_23), one *cathepsin L* (*etcpl*, on contig NODE_2923_length_1315_cov_12.253232), and three *cathepsin Cs* (*etcpc1*, ETH_00019755 on Supercontig_2; *etcpc2*, ETH_0005000, on contig NODE_22022_length_2554_cov_8.124119; and *etcpc3*, ETH_00001590, ETH_00001595, ETH_00001600, on Supercontig_115) were identified using BLASTP to search the *E. tenella* genome database (http://www.genedb.org/Homepage/Etenella) using, as queries, the putative homologs described in other apicomplexan parasites, namely *T. gondii* and *P. falciparum*.

**Figure 1:**
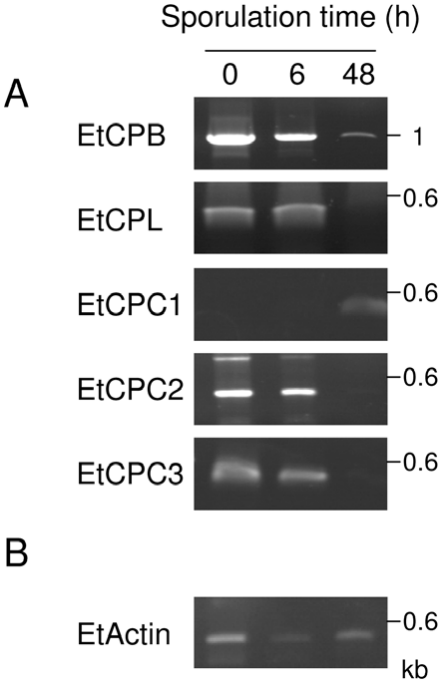
Expression profile of CPs in *E. tenella*. (A) Total RNAs from unsporulated oocysts (0), oocysts in sporulation for 6 h (6) and fully sporulated oocysts (48) were extracted and RT-PCR were performed using specific primers to *etcpb, etcpl, etcpc1, etcpc2* and *etcpc3*. (B) The single-copy *actin* gene was amplified in parallel as a control (*etactin*). The down regulation of *etactin* during sporulation has been previously described [17]. The RT-PCR products were resolved on a 0.7% agarose gel stained with ethidium bromide. The band observed at 0.8 kb with primers specific to *etcpc2* and total RNAs from unsporulated oocyst (0) is a nonspecific amplification product.

To begin addressing the biological roles of these proteases, we examined their expression patterns by RT-PCR on total RNAs from three different time points of oocyst sporulation: unsporulated (0 h of sporulation); partially sporulated (6 h of sporulation); and fully sporulated (48 h) (Figure 1). Expression of actin was assessed as a control and, whilst expressed at every time point tested, it was noticeable that actin expression appeared to be downregulated significantly during sporulation with expression being upregulated again in fully sporulated oocysts (Figure 1). This is totally consistent with previous findings [17]. A cathepsin L (EtCPL) and two cathepsin Cs (EtCPC2 and EtCPC3) were mainly expressed in unsporulated oocysts and partially sporulated oocysts. In contrast, a third cathepsin C (EtCPC1) appeared to be expressed in fully sporulated oocysts only. A cathepsin B-like enzyme (EtCPB) was the only protease expressed throughout sporulation, though it was more strongly expressed in unsporulated oocysts and at the earliest time point of sporulation (Figure 1).

### Biochemical characterization of a cathepsin B-like activity in oocyst lysates

**Figure 2:**
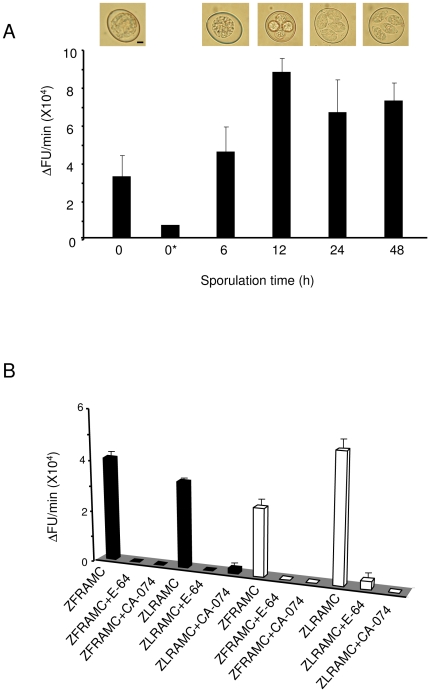
Biochemical activities of CPs throughout sporulation. (A) Activities detected on Z-FR-AMC. Lysates of oocysts (1 mg/ml) taken at 0, 6, 12, 24 and 48 h after the beginning of sporulation were incubated with the substrate Z-FR-AMC (10 µM). The morphology of oocysts (under light microscopy) throughout the course of sporulation is shown. The scale bar represents 2 µm. (B) Activities detected on Z-FR-AMC and Z-LR-AMC in presence of the global cysteine protease inhibitor E-64 or the human cathepsin B specific inhibitor CA-074. Lysates (1 mg/ml) of oocysts taken at 0 h (black bars) and 48 h (white bars) after commencement of sporulation were pre-incubated with the inhibitors before adding the substrates. The data represents two independent experiments.

To analyze the putative activities of CPs during sporulation, we performed a biochemical assay on oocyst lysates taken 0, 6, 12, 24, 36 and 48 h after the commencement of sporulation. Each lysate was incubated with the fluorescent substrates, Z-FR-AMC, Z-LR-AMC (Figure 2). Z-FR-AMC and Z-LR-AMC are substrates for both cathepsin B and cathepsin L-like enzymes, shown to be expressed by *E. tenella* (Figure 1). These dipeptides are cleaved by active cysteine endopeptidases at the C-terminus of the arginine residue resulting in the release of AMC. Free AMC emits a fluorescent signal that can be quantified by spectrofluorimetry [18], [8], [19]. Proteolytic activities towards Z-FR-AMC that corresponded to active cysteine proteases were detected in each oocyst lysate along the oocyst maturation. The emitted fluorescence was linear with time. These activities increased until 12 h of sporulation to reach a plateau that persisted in fully sporulated oocysts (Figure 2A). Pre-incubation of unsporulated oocyst lysate with the broad-spectrum cysteine protease inhibitor, E-64, resulted in a greatly reduced signal, thus confirming that cysteine proteases were responsible for the activities observed on Z-FR-AMC. Similarly, unsporulated and sporulated oocysts showed activities on Z-LR-AMC and these activities were inhibited by E-64 (Figure 2B). These data demonstrated that a single or several CPs were active during sporulation, suggesting a role in this crucial step of the *Eimeria* life cycle. Since the analysis of the expression profile of *Eimeria* CPs revealed that an mRNA encoding a cathepsin B-like protease was expressed both in unsporulated and sporulated oocysts, we investigated the contribution of this enzyme to the observed activities on Z-FR-AMC and Z-LR-AMC (Figure 2B). Unsporulated and sporulated oocyst lysates were pre-incubated with CA-074, a specific inhibitor of human cathepsin B, before adding the substrates. The inhibitor completely abolished the activities on both substrates in each lysate. In addition, we detected an activity of the unsporulated oocyst lysate on the specific cathepsin B-like substrate Z-RR-AMC (ΔFU/min = 6700 +/− 1500). Taken together, these data demonstrated that a cathepsin B-like protease was expressed, and was the only activity detected throughout sporulation.

### Activity profile of CPs throughout sporulation

**Figure 3:**
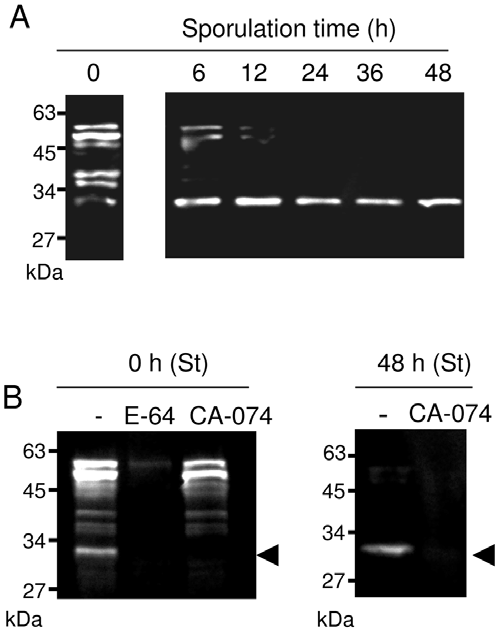
Profile of active CPs throughout sporulation. (A) Lysates of unsporulated oocysts (0) and oocysts in sporulation for 6, 12, 24, 36 and 48 h were incubated with the probe Biot-LC-LVG-CHN_2_. The complexes were revealed by Western blot using streptavidin peroxidase. (B) Lysates of oocysts obtained at 0 h or 48 h of sporulation time (St) were pre-incubated or not (−) with E-64 (28 µM) or CA-074 (100 µM) before addition of Biot-LC-LVG-CHN_2_. The interaction between the probe and CP catalytic sites was detected by Western blot using streptavidin peroxidase. The arrowhead indicates the activity corresponding to the *E. tenella* cathepsin B (33 kDa).

We performed a biochemical assay based on the interaction between proteases and the activity-based probe Biot-LC-LVG-CHN_2_. This compound is derived from the N-terminal substrate-like sequence of human cystatin and has been shown previously to interact specifically with the nucleophilic cysteine of the catalytic sites of CPs expressed by *Trypanosoma cruzi* and *P. falciparum*, cruzipain or falcipains, respectively [20], [21]. The biotin group allows the direct detection of CPs in the presence of streptavidin peroxidase. Lysates of unsporulated, partially sporulated and fully sporulated oocysts were incubated with Biot-LC-LVG-CHN_2_ and the complexes formed with *Eimeria* CPs were analyzed (Figure 3). Up to eight main complexes were revealed in the lysates of unsporulated oocysts. They corresponded to proteins of 33, 35, 36, 38, 44, 46, 50 and 55 kDa. Remarkably, 6 h after the beginning of sporulation, the majority of signal intensities decreased dramatically except for the one corresponding to the 33 kDa band (Figure 3A). Moreover, this unique band of a constant intensity persisted until the completion of sporulation. Next, we used specific inhibitors to identify the proteases revealed by the probe (Figure 3B). Addition of E-64 to the unsporulated oocyst lysate, before incubation with the probe, resulted in the complete or drastic loss of the signals demonstrating the specific interaction between Biot-LC-LVG-CHN_2_ and CPs expressed by *E. tenella*. To determine if cathepsin B activity was detected by the probe, unsporulated and sporulated oocyst lysates were pre-incubated with CA-074 before adding the probe Biot-LC-LVG-CHN_2_. Addition of CA-074 specifically inhibited the 33 kDa corresponding activity. This data revealed that the 33 kDa band that persisted throughout sporulation corresponded to *E. tenella* cathepsin B.

### Amino acid alignment of *E. tenella* cathepsin B with parasitic, mammalian and bird cathepsin B

**Figure 4:**
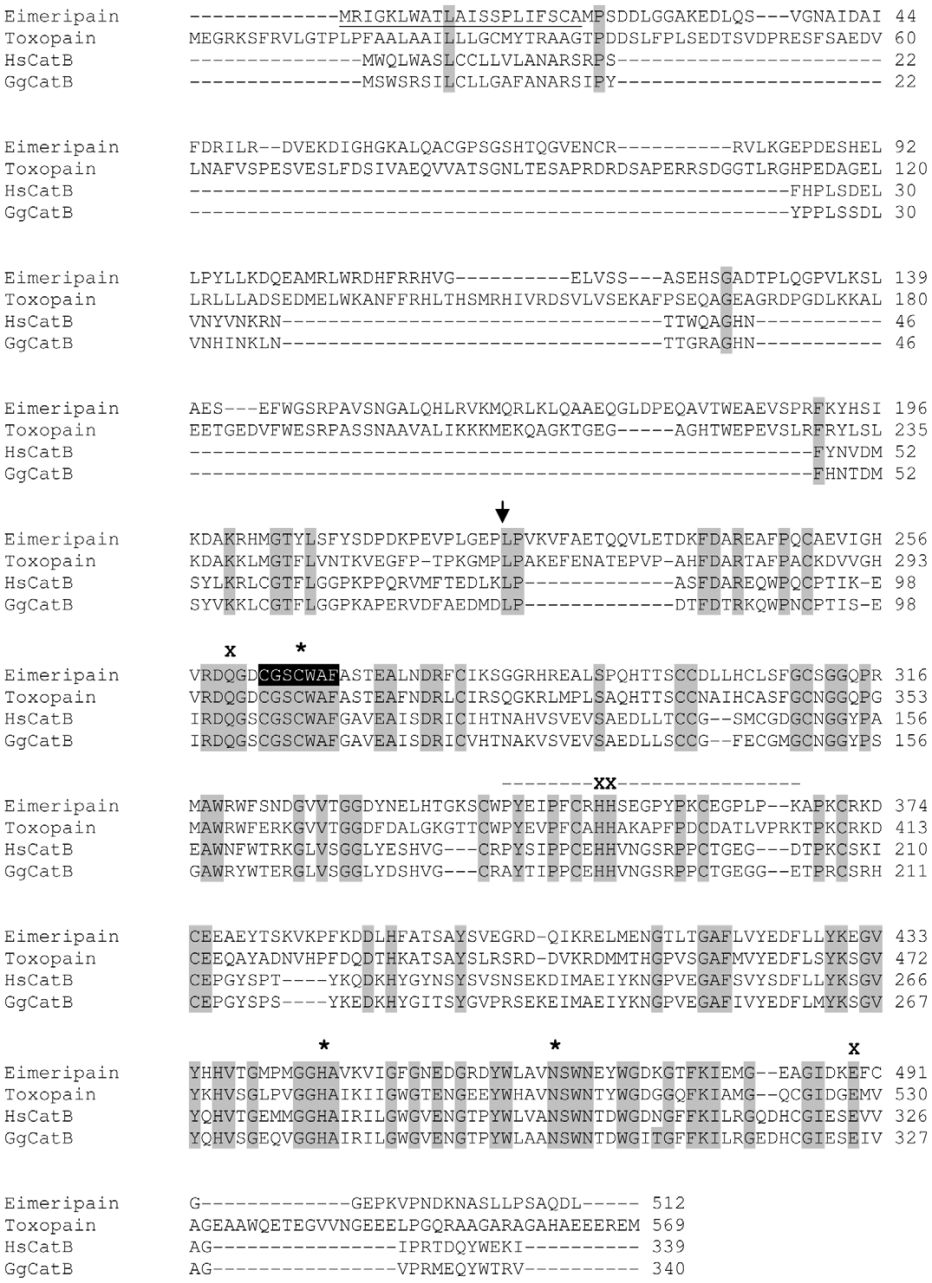
Sequence alignment of the pro-protein of the *E. tenella* cathepsin B. An alignment of amino acid sequences of the *E. tenella* cathepsin B with cathepsin B expressed by *T. gondii* (toxopain-1, Accession n° AAL60053), *Homo sapiens* (HscatB, AAC37547.1) and *Gallus gallus* (GgCatB, AAA87075.1) is shown. The predicted signal peptide, according to SignalP (www.expasy.org) for the *E. tenella* cathepsin B is underlined. Identical residues are shaded in gray. The arrow indicates the probable cleavage site separating the pro- from the catalytic domain. Asterisks indicate the conserved essential catalytic triad residues. The predicted conserved occluding loop is shown with a dashed line. The conserved peptide sequence containing the catalytic Cys266 is highlighted in black. The characteristic aminoacids of cathepsin B like proteases are indicated by a cross and correspond to Gln260 of the oxyanion hole, the two His352-His353 of the occluding loop, and Glu489, at the base of the S2 pocket.

We cloned and sequenced the gene encoding the *E. tenella* cathepsin B. An alignment of the amino acid sequence of this enzyme with toxopain-1 (cathepsin B expressed by *T. gondii*), human cathepsin B and chicken cathepsin B was performed (Figure 4). The *E. tenella* cathepsin B is a 512 amino acid protein composed of a signal peptide (from Met1 to Ala21) identified by signalP, a predicted prodomain (from Met22 to Pro223) and a catalytic domain (from Leu224 to Leu512). The prodomain is much longer (214 amino acids) than mammalian and bird cathepsin B, as also reported for toxopain-1 [3]. The *E. tenella* cathepsin B contains the two archetypal residues (Cys266 and His445) that form the thiolate-imidazolium ion pair and are involved in the nucleophilic attack of the peptidyl substrate as well an asparaginyl residue (Asn465) reported to critically support the catalytic mechanism. C266 is embedded in the highly conserved peptide sequence, CGSC266WAF. Specific cathepsin B motifs are also found in the *E. tenella* sequence including the G308CXGG motif as well as the residues that form the occluding loop, which is characterized by two adjacent histidine residues (His352, His353) and encompasses amino acid residues Pro344 to Cys371. In addition to its endopeptidase activity, the presence of the occluding loop confers, specifically to cathepsin B, a dipeptidyl carboxypeptase activity [22] as well as a poor inhibitory potential towards kininogens compared to stefins and cystatins [23].

### Molecular modelling of *E. tenella* cathepsin B

**Figure 5:**
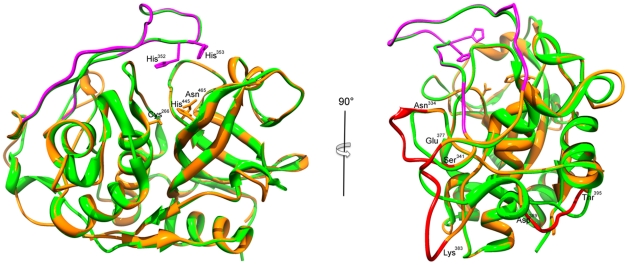
Ribbon diagram superposition of the catalytic domains of the *E. tenella* cathepsin B and human cathepsin B. The X-ray crystallographic structure of the human cathepsin B (pdb 1 gmy) is colored in green and the proposed structure of homology-based model of the *E. tenella* cathepsin B is in orange. The catalytic triad residues (Cys266, His445 and Asn465) are depicted in the *ball-and-stick* representation. On the left panel, the occluding loop is represented in purple with the two adjacent histidine residues (His352, His353) in the *ball-and-stick* representation. On the right panel, the surface exposed loops specific to *E. tenella* cathepsin B are in red. The residues delimitating the loops: Asn334-Ser341, Glu377-Lys383 and Asp389-Thr395 are shown.

The structure of the *E. tenella* cathepsin B was determined by molecular replacement using the structure of the catalytic domain of human cathepsin B, with which it shares 43.1% sequence identity. Eimeripain exhibits the typical papain-like fold that is representative of the cysteine cathepsin family (Clan CA, family C1). It is composed of two domains, referred to as the left (L-) and right (R-), in accordance with the standard view and nomenclature (See for review, [24]), [25]. Thus, the left domain has three helical regions, the right domain is composed of a barrel of six strands, which includes a shorter α–helical motif, and the catalytic triad is housed in a cleft separating the two domains, with Cys266 located in the left domain and His445 and Asn465 (*E. tenella* numbering) provided by the right domain (Figure 5). The *E. tenella* structure clearly resembles cathepsin B-type peptidases with the insertion of the so-called occluding loop, between the conserved Pro-Tyr345 motif and Cys371. The loop is characterized by two adjacent histidine residues (His352, His353), which block the active site cleft on the primed binding site (beyond S'2 subsite) and are responsible for the dipepdityl carboxypeptidase activity of cathepsin B. A comparison of the homology-based model with human cathepsin B shows a high degree of similarity (rmsd 0.49 Å for 245 Cα atoms). The major structural changes in backbone superposition correspond to additional residues and localize at the surface exposed loops including residues Asn334-Ser341, Glu377-Lys383 and Asp389-Thr395. We have, based on its susceptibility to specific inhibitors, its sequence homology and the predicted 3D structure, named the *E. tenella* cathepsin B, eimeripain.

### Localization of eimeripain in oocysts and sporozoites

**Figure 6:**
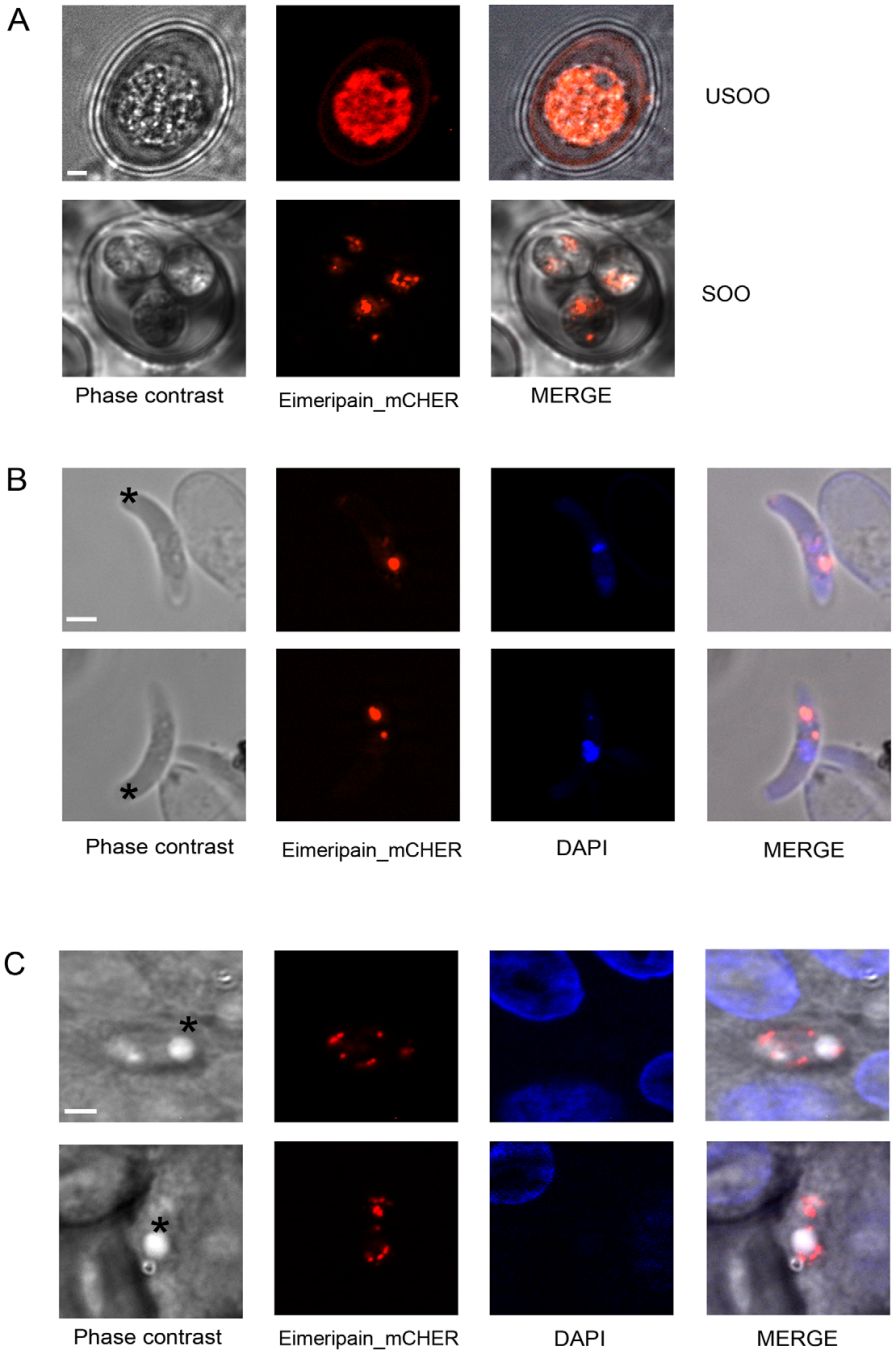
Localization of eimeripain_mCHER within *E. tenella*. Sporozoites were transfected with the plasmid pEimerip/Eimerip_mCherry and directly inoculated into chickens via the cloaca to complete the *Eimeria* life cycle *in vivo*. Seven days post-inoculation, oocysts were harvested from the caeca. mCherry+parasites (red) were observed with a Zeiss Axiovert 200 microscope equipped with the Apotome imaging system. (A) Localization of eimeripain_mCHER in unsporulated oocysts (USOO) and sporulated oocysts (SOO). (B) Localization of eimeripain_mCHER in extracellular sporozoites. Nuclei of sporozoites were stained with DAPI (blue). Two examples of eimeripain_mCHER expressing sporozoites are shown. (C) Localization of eimeripain_mCHER in intracellular sporozoites. Infected MDBK cells were fixed and observed 24 h post invasion by recombinant sporozoites. Nuclei of host cells are detected with DAPI (blue). Two examples of intracellular eimeripain_mCHER expressing sporozoites are shown. The posterior refractile body is indicated by an asterisk. The scale bar represents 2 µm.

The recent advances in the development of transgenesis in *E. tenella* allows, for the first time, consideration of knock-in and knock-out strategies to determine the function of genes in the biology of the parasite [26], [27]. To localize eimeripain within the parasite, we used a stable transfection strategy to make the parasite express a tagged version of eimeripain (Figure 6). The fusion protein, eimeripain_mCHER, consisted of eimeripain fused, in frame, to its C-terminus with the fluorescent protein mCherry (red). The endogenous promoter of eimeripain was chosen to drive the expression of eimeripain_mCHER to maintain regulation of expression similar to that observed with the endogenous *eimeripain* gene. In a first set of experiments, we looked at the localization of the fusion protein within the unsporulated and sporulated oocysts (Figure 6A). Recombinant unsporulated oocysts expressed eimeripain_mCHER and the protein was uniformly distributed in the zygote. In contrast, in the sporulated oocysts, eimeripain_mCHER had a punctate distribution within sporozoites. In a second set of experiments, we analyzed the localization of the tagged protease within extracellular (Figure 6B) and intracellular (Figure 6C) sporozoites. In both conditions, eimeripain_mCHER was observed as several dots in the parasite bodies.

### Role of eimeripain in cell invasion by sporozoites

**Figure 7:**
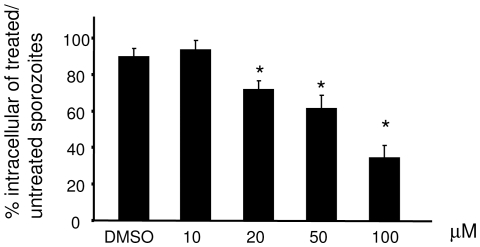
Effect of CA-074 Me on MDBK cell invasion by *E. tenella* sporozoites. Purified sporozoites were incubated with 10 to 100 µM of CA-074ME, a specific cathepsin B inhibitor permeable to membranes or with 1% DMSO. Sporozoites were then washed and incubated with MDBK cells. Infected cells were fixed and intracellular parasites were detected by IF using specific sera against sporozoites, and counted. The data represent three independent experiments. * denotes significant differences at p<0.05 in the capacity of CA-074 Me- treated parasites to invade cells compared to the control.

We showed that eimeripain was revealed as a 33 kDa band by the probe Biot-LC-LVG-CHN_2_ both in the unsporulated and sporulated oocysts (Figure 3). This size was expected for the active form of the protease, suggesting a role during sporulation but also, potentially, in the first steps of the infectious process (Figure 3). Therefore, we addressed the potential role of eimeripain in cell invasion by sporozoites. The invasion assay was performed with *E. tenella* sporozoites pre-treated or not with CA-074 Me, a cell permeable inhibitor of cathepsin B (Figure 7). Addition of the inhibitor significantly (P<0.05) impaired the capacity of the sporozoites to invade MDBK cells with a 63% inhibition of invasion in the presence of 100 µM of CA-074 Me. This phenotype was not due to a lethal effect of CA-074 Me since in an acridine orange/ethidium bromide viability assay, inhibitor-incubated-sporozoites had statistically similar viability to that seen in inhibitor-free samples. Thus, the mean viability (n = 2) of sporozoites incubated for 1 hr at 41°C with 0, 10, 20, 50 or 100 µM of CA-074 Me was 94%, 84%, 88%, 90% and 84%, respectively; one-way analysis of variance showed that there were no significant differences in the mean values of these different treatment groups with a P value of 0.7355, an F value of 0.506 and an R-squared of 0.2881. Consequently, these data indicated that eimeripain plays an important role in cell invasion by sporozoites of *E. tenella*.

## Discussion

CPs are major virulence factors for protozoan parasites. They are implicated in key steps of infectious processes making them attractive therapeutic targets (for review see [28]). In this study, we characterized CPs expressed by *E. tenella*, one of the most virulent species of *Eimeria* in chickens. By blasting the *E. tenella* genome (http://www.genedb.org/Homepage/Etenella), we found five genes encoding for putative cysteine proteases: one homolog to cathepsin B (eimeripain); one to cathepsin L (EtCPL); and three to cathepsin C (EtCPC1, EtCPC2 and EtCPC3). The gene predictions did not allow us to identify the start and stop codons for all genes; however, we were able to determine their expression profiles by using specific primers for conserved regions and were able to show that all these predicted genes are transcribed. Although five cysteine protease genes were identified in the genome of *E. tenella*, the probe Biot-LC-LVG-CHN_2_ detected up to eight polypeptides in the unsporulated oocyst. The probe might have detected other cysteine proteases whose genes are specific to the *Eimeria* genus or multiple forms of the same protease.

Only one mRNA of the five genes coding for cathepsins – that encoding EtCPC1 – is expressed exclusively in the fully sporulated oocysts. However, EtCPC1 is not labelled by Biot-LC-LVG-CHN_2_ at this stage. While it is possible that the probe did not label the active form of EtCPC1, an alternative explanation is that EtCPC1 may remain in its zymogen form before maturation and activation in further steps of the parasite life cycle. Consistent with this latter hypothesis, TgCPC1, the homolog of EtCPC1 in *T. gondii*, is associated with the parasitophorous vacuole and is involved in intracellular survival of the parasite [9]. Accordingly, EtCPC1 may similarly remain inactive in oocysts, and then be activated during intracellular parasite development, which starts several hours post invasion of epithelial cells by sporozoites [29], [30].

Three of the five genes coding for cathepsins – the cathepsin L and two of the cathepsin Cs (EtCPC2 and EtCPC3) – are expressed in unsporulated oocysts and at the earliest time point of sporulation analyzed in this study (6 h) but not at later time points or in fully sporulated oocysts. This suggests a role(s) in the initiation of sporulation, a component of the parasite's sexual cycle where meiotic division of the diploid zygote, within the unsporulated oocysts, leads to the production of four haploid sporoblasts. However, this idea awaits demonstration since all of the detected protease activity against Z-FR-AMC and Z-LR-AMC in oocyst lysates can be inhibited by the cathepsin B-specific inhibitor, CA-074. Z-FR-AMC and Z-LR-AMC are specific substrates for CPs with endopeptidase activities (i.e., cathepsin B and cathepsin L), so cathepsin Cs, which exhibit dipeptidyl aminopeptidase activities, are not expected to cleave these substrates. However, the same argument cannot account for the absence of cathepsin L-specific activity on Z-FR-AMC or Z-LR-AMC; this may reveal a specific biochemical property of EtCPL or indicate that it is maintained in an inactive pro-form in unsporulated oocysts.

We identified a single gene coding for a cathepsin – a cathepsin B – that is expressed throughout sporulation. Activity associated with this enzyme corresponds to a 33 kDa protein band detected in all oocyst stages by the probe Biot-LC-LVG-CHN_2_. The *E. tenella* cathepsin B shares 43.1% sequence identity with the catalytic domain of human cathepsin B and 53% identity with toxopain-1, the cathepsin B from the related apicomplexan parasite, *T. gondii* [3]. The *E. tenella* cathepsin B has the active triad sequence of cysteine, asparagine and histidine and, like toxopain-1, contains a characteristic occluding loop sequence and has a relatively long pro-domain. Homology modelling reveals that the overall structure of the catalytic domain is very similar to that of human cathepsin B, which in turn is very similar to that predicted for toxopain-1 [3]. We have, therefore, named the *E. tenella* cathepsin B, eimeripain.

Studies performed on trafficking of human cathepsin B have shown that a C-terminus GFP fusion of the protease is correctly trafficked and targeted to lysosomal compartments in thyroid epithelial cells [31]. We therefore decided to study the localization of eimeripain by transfecting sporozoites with a version of the protease that has its C-terminus fused to the red fluorescent protein, mCherry [26], [27]. To our knowledge, our work represents the first use of transfection to study the localization of a protein expressed by *E. tenella*. Unsporulated oocysts express the fusion protein, eimeripain_mCHER, uniformly in the zygote. In contrast, eimeripain_mCHER has a punctated distribution in sporozoites. This localization is similar to that described for cathepsin L in *T. gondii* (TgCPL). TgCPL is associated with endocytic organelles, most particularly a newly reported vacuolar compartment at the apical region of tachyzoites that undergoes dynamic fragmentation during *T. gondii* replication [32], [33]. It is not yet known whether such vacuolar, lysosomal-like compartments are also present in sporozoites of *Eimeria*.

TgCPL has been shown to be important for proteolytic maturation of pro-proteins targeted to the micronemes [32] and its inhibition severely impairs cell invasion [5]. Indeed, invasion of target cells by apicomplexan parasites is a conserved mechanism involving the sequential secretion of apical organelles involved in cell attachment (micronemes) and penetration (rhoptries) [34], [35]. Eimeripain, in contrast to toxopain-1, which is found in the rhoptries [3], does not appear to localize in these compartments (Figure 6). However, the inhibition of this protease by CA-074 Me inhibits cell invasion by *E. tenella* (Figure 7). The effect of CA-074 Me on cell invasion by sporozoites of *E. tenella* may be indirect; CA-074 Me could inactivate eimeripain and impair the proteolytic processing of substrates necessary for proper cell invasion to occur, as observed with TgCPL [32]. Eimeripain, similarly to TgCPL, may be responsible for the maturation of one or several substrates belonging to these compartments. However, it should also be noted that, since the active form of this cathepsin B is also expressed in unsporulated oocysts, the protease may also have a specific function in sporulation that remains to be determined.

In model apicomplexan parasites, CPs have been implicated mainly in key facets of asexual cell biology. This is reinforced in our study where we show that eimeripain plays a role in cell invasion by *E. tenella* sporozoites. On the other hand, another implication of our work is that CPs may play an as yet undescribed role in sporulation. There is currently only circumstantial evidence that proteases are involved in sporulation [13], [14], [15], [16] and our descriptions of the relative timings of expression of different CPs during sporulation adds to this body of evidence. However, unsporulated oocysts undergo major morphological changes during sporulation and such remodelling typically involves proteases. With the advent of gene transfection strategies, as described by us and others [26], [27], *Eimeria* becomes a particularly suitable model to study the molecular mechanisms involved in sporulation. The large number of oocysts shed are easily purified, allowing performance of proteomic and molecular studies that are essential to understand the mechanisms involved in oocyst development; similar studies in other apicomplexans are substantially more difficult. The determination of the role of *E. tenella* CPs expressed at these stages may help the discovery of new targets aimed at preventing dissemination of the parasite.

## Materials and Methods

### Parasites and cells

Groups of outbred PA12 chickens of 4 to 6 weeks old of age were inoculated orally with 10^4^ sporulated oocysts of *E. tenella* Wis strain [36]. Seven days post-inoculation, unsporulated oocysts were harvested from infected caeca, purified and kept at 4°C with 1 mM sodium dithionite (Sigma Aldrich) to prevent sporulation until needed [37]. Care and euthanasia of animals were practised according to national ethic guidelines. For the sporulation time course experiments, sodium dithionite was removed and purified unsporulated oocysts were incubated at various times at 25°C in 2% potassium dichromate and processed immediately for further biochemical analysis [37]. Sporozoites were obtained after breaking sporulated oocyst walls with glass beads and incubated in the excystation medium (biliary salts 0.5%, trypsin 0.25% diluted in PBS pH 7.6) at 41°C for 75 min. Sporozoites were purified by a two step filtration protocol, first on cotton and then on polycarbonate filters (5 µm) (GE Water & Process Technologies).

Madin-Darby bovine kidney (MDBK) cells [38] were propagated in Ham's F12 medium containing 5% foetal calf serum, 2 mM glutamine, 10 UI/ml penicillin, 10 µg/ml streptomycin (Lonza).

### Ethics statements

Experimental protocols were designed in compliance with French law (Décret 2001-464 29/05/01) concerning the use of laboratory animals. Care and euthanasia of animals were practised according to national ethic guidelines and approved by the ethics committee of the Région Centre (CL2007-36). The authors are committed to the principals of the 3Rs: Reduction, refinement and replacement of experimental animals.

### Identification of CP encoding genes in *E. tenella* genome

*E. tenella* genome sequences and gene models were downloaded from http://www.genedb.org/Homepage/Etenella The genome of *E. tenella* Houghton was produced by the Parasite Genomics Group at the Wellcome Trust Sanger Institute (http://www.sanger.ac.uk/research/projects/parasitegenomics/) and has been provided prepublication. BLASTP search was performed in GeneDB using as queries CPs described in *T. gondii* including one cathepsin B, toxopain-1 (AAL60053), one cathepsin L, TgCPL (ABD64744), and three cathepsin Cs, TgCPC1 (AAZ15654), TgCPC2 (AAZ15655) and TgCPC3 (AAZ15656); and *P. falciparum* including falcipain-1 (AAN37166), falcipain-2 (AAK06665), falcipain-2′ (AAX77225), falcipain-3 (AAF77192), DPAP1 (AAN35758), DPAP2 (AAN36542) and DPAP3 (XP_001351359). CP genes in *Eimeria* have been named after their closest homologs in *T. gondii*.

### RNA extraction and RT-PCR on oocyst lysates using specific CP primers

Pellets of oocysts (5×10^7^), taken at different time after the commencement of sporulation, were vortexed with 0.5 mm glass beads until the total destruction of oocyst walls. Total RNAs were obtained by treatment of broken oocysts with TRIzol (1 ml) (Invitrogen) for 5 min at room temperature, extraction with 20% chloroform, and precipitation with 50% isopropanol. RNAs were resuspended in nuclease-free water to a final concentration of 1 mg/ml. Aliquots of 1 µg of total RNAs were used to reverse transcribe total mRNAs using a Poly dT primer and reverse transcriptase II (Invitrogen). Segments of cDNAs were amplified using specific primers to *eimeripain*, 5′-CTGGGGAAGCCGGCCAGCTGTTAGC-3′ and 5′-CGTTCCAGCTGTTCACAGCTAGCCAG-3′, to *etcpl*, 5′-CAACCAACAAGGTCACTCTTAC-3′ and 5′-CCCTCGAGGGCCCCCGTGCTCG-3, to *etcpc1*, 5′-CGCCAAAGGCTACGGCTACGTGG-3′ and 5′-CCGCGAGTGAGGTCGGGGTCAATG-3′, to *etcpc2*, 5′-CCAAATCAGGGCGACTGCGGCTCTTG-3′ and 5′-GCCCCCAACATAATACCATTTGGAAAC-3′, to *etcpc3*, 5′-CGCTCAGGAGTACAACTACGTGGGTGG-3′ and 5′-GCTGCTGCACAAGCAGCGCTGCTCTGCC-3′, to *actin*, 5′-ATCTTCATCGTAGACGGAGCCAG-3′ and 5′-GTGTGTCCCACACTGTTCCTATC-3′. The reaction conditions for RT-PCR were 95°C for 1 min, 58°C for 30 s and 72°C for 1 min for 30 cycles using the Go Taq DNA polymerase (Promega). Amplification products were loaded onto a 0.7% agarose gel stained with ethidium bromide.

### Activities of CPs in oocyst lysates

Oocysts (5×10^7^) were removed at different time after the commencement of sporulation and lysates were obtained by vortexing pellets with 0.5 mm glass beads until the total destruction of oocyst walls [37]. Pellets were resuspended in buffer A (sodium acetate 100 mM, pH 5.5, in the presence of 1 mM phenylmethanesulfonylfluoride (PMSF), 0.04 mM pepstatin A, 1 mM methylmethane thiosulfonate (MMTS) and 1 mM EDTA) to obtain a final concentration of proteins in oocyst lysate of 1 mg/ml as determined using the Bradford protein assay (Biorad). Beads were removed and lysates were sonicated twice for 30 s, amplitude 40 (Bioblock scientific, Vibracell 75455), centrifuged for 10 min at 13000 rpm at 4°C and the supernatant kept at −80°C for future biochemical experiments to detect CP activities using either fluorescent dipeptides or the probe, Biot-LC-LVG-CHN_2_ [20]. To detect endopeptidase CP activities using fluorescent dipeptides, lysates were preincubated or not with 28 µM E-64 or 100 µM L-3-*trans*-(propylcarbamyl)oxirane-2-carbonyl)-L-isoleucyl-L-proline (CA-074) for 10 min at 30°C in buffer A and 2 mM DTT before addition of either 10 µM Z-FR-AMC (benzyloxycarbonyl-Phe-Arg-(7-Amido-4-methylcoumarin)), 10 µM Z-LR-AMC ((benzyloxycarbonyl-Phe-Arg-(7-Amido-4-methylcoumarin)) or 10 µM Z-RR-AMC ((benzyloxycarbonyl-Arg-Arg-(7-Amido-4-methylcoumarin)) (Bachem). Activities were monitored on a spectrofluorimeter (Photon Technology International) with λ_excitation_ = 350 nm, λ_emission_ = 450 nm. Independently, lysates were preincubated or not for 1 h at 25°C with 100 µM CA-074 before addition of 20 µM of the cystatin-derived activity-based probe Biot-LC-LVG-CHN_2_ for 1 h at 25°C in buffer A with 2 mM DTT. Samples were loaded on a 10% SDS-polyacrylamide gel, transferred onto a nitrocellulose membrane (GE healthcare) and tagged active CPs were revealed by Western blot using streptavidin coupled to peroxidase (1/3000). Detection was performed using the Supersignal West Pico Chemiluminescent Substrate (Thermo Scientific).

### Cloning of eimeripain cDNA and construction of pEimerip/Eimerip_mCherry localization vector

By blasting the *E. tenella* genome against toxopain-1 (cathepsin B in *T. gondii*), we obtained a hit with gene ID ETH_00003570 (E value = 1.1e-122). Primers were designed upstream from the predicted start site (fETCPB3: 5′-GCCAACCACAGCGCAGCTCCGAGGTG-3′) and downstream from the predicted STOP codon (rETCPB3: 5′-GCCGCGGTTAGCCGCAACTTCGACC-3′) to amplify the cathepsin B gene (*eimeripain*) from *E. tenella* sporozoite cDNAs. The amplification product was sequenced, which confirmed the sequence prediction of ETH_00003570. To localize eimeripain within the parasite, we constructed a vector allowing the expression of an eimeripain_mCHER fusion under the control of the *eimeripain* promoter. Since *eimeripain* does not contain any intron, we used genomic DNA to amplify a 3 kb region with primers fPrEtCPBMlu and rEtCPBSal. The amplification product, MluI-PrEtCPB/EtCPB-SalI, started 1 kb upstream from the start codon of *eimeripain* (this region is hypothesized to contain the endogenous promoter of *eimeripain*, as commonly observed in *T. gondii*) until the last amino acid codon of *eimeripain*. MluI-PrEtCPB/EtCPB-SalI was cleaved by MluI and SalI and cloned into the mCherry core construct-1 kindly provided by Dr Damer Blake [26]. The mCherry core construct-1 contains the promoter of the microneme protein-1 (Mic-1), which drives the expression of the fluorescent protein mCherry (λExcitation = 587 nm; λEmission = 610 nm). mCherry core construct-1 was cleaved by MluI and SalI to remove the *mic-1* promoter, which was replaced by the 3 kb MluI-PrEtCPB/EtCPB-SalI insert, leading to the vector pEimerip/Eimerip_mCherry in which eimeripain is C-terminally fused in frame with mCherry. The fusion protein eimeripain-mCHER is expressed under the control of the endogenous *eimeripain* promoter.

#### Alignment analysis and molecular modelling of eimeripain

The protein sequences for pro-eimeripain (Accession n° JN641867), pro-toxopain-1 (AAL60053), pro-human cathepsin B (AAC37547.1) and pro-chicken cathepsin B (AAA87075.1) were aligned by using CLUSTALW2 (http://www.ebi.ac.uk/Tools/msa/clustalw2/). An automated comparative protein modelling program, SWISS-MODEL, was used to predict the 3D structure of the eimeripain catalytic domain [39]. Human cathepsin B served as a template protein (pdb accession nb: 1 gmy). The reliability of the generated model was evaluated by QMEAN4 scoring function. Molecular illustrations were prepared using UCSF Chimera (UCSF Chimera-a visualization system for exploratory research and analysis [40].

### Localization of eimeripain within parasites

Freshly excysted sporozoites were resuspended in complete cytomix buffer (10 mM K_2_HPO_4_/KH_2_PO_4_, pH 7.6, 120 mM KCl, 0.15 mM CaCl_2_, 25 mM HEPES, 2 mM EGTA, 5 mM MgCl_2_, 2 mM ATP and 5 mM glutathione) as described previously [26]. pEimerip/Eimerip_mCherry vector was linearized with ScaI (2 U) and 20 µg added to 100 µl of cytomix resuspended sporozoites (2×10^6^ parasites) for REMI transfection [26]. Electroporation occurred in a gene pulser electroporator (Biorad) using 1000 V, 25 µF in a 2 mm electroporation cuvette. To obtain stable transfectants, electroporated sporozoites were resuspended in Ham's F12 medium with 3% FBS (2 ml) and immediately inoculated into five PA12 chickens (2×10^5^ transfected sporozoites per animal) via the cloacal route as described previously [26]. Seven days post-inoculation, recombinant oocysts were harvested from the caeca and kept at 4°C or incubated at 25°C for 48 h to obtain sporulated oocysts.

Purified recombinant sporozoites were fixed in 2.7% paraformaldehyde (PFA) and air dried on a glass slide or incubated with MDBK cells for 24 h. Extracellular parasites and infected cells were fixed in 2.7% PFA for subsequent immunofluorescence analysis. Cell and parasite nuclei were fixed and stained with 1.5 µg/ml of DAPI (Vectashield). Fluorescence imaging was done using a Zeiss Axiovert 200 microscope equipped with the Apotome imaging system. Images were generated and analyzed by using the Axiovision Software (Carl Zeiss SA).

### *E. tenella* viability assay

Viability of extracellular *E. tenella* sporozoites pre-incubated with CA-074 methyl ester (CA-074 Me), a specific cell-permeable inhibitor of cathepsin B, was assessed using acridine orange and ethidium bromide, as described previously [41], [42]. Acridine orange is able to penetrate all parasites and stains nucleic acids green, whereas ethidium bromide, which binds to nucleic acids and fluoresces orange, is only able to enter parasites once the integrity of the cell membrane is compromised. Sporozoites (4×10^5^) were incubated with various concentration of CA-074 Me for 1 h at 41°C. Parasites were washed twice in PBS and acridine orange and ethidium bromide were added at 0.1 µg/ml. Extracellular parasites were visualized and counted using an Olympus BX41 microscope. The percentage of non viable parasites was determined as the ratio of red dead parasites *versus* the entire green population ×100.

### Invasion assay

Freshly excysted purified sporozoites (2×10^5^) were incubated with non lethal concentrations of CA-074 Me for 1 h at 41°C in 500 µl of Ham's F12. Sporozoites were washed twice in PBS and incubated with MDBK cells in 24 well plates with coverslips at the bottom, (multiplicity of infection of one), for 1 h at 41°C, 5% CO_2_. Infected cells were washed and fixed in 2.7% PFA. Monolayers were then permeabilized with 0.1% Triton X-100, and intracellular parasites were labelled with a mouse anti-sporozoite antibody (1/2000) followed by incubation with a secondary anti-mouse antibody coupled to Alexa 594 (1/1000) (red fluorescence). Monolayers were mounted in Vectashield containing 1.5 µg/ml of DAPI (Clinisciences, France) to label nuclei. Infected cells were examined using a Zeiss Axiovert 200 microscope. The percentage of intracellular parasites was determined as the number of intracellular parasites (red) for 100 MDBK cells (blue nuclei). More than 200 cells were counted for each condition in three independent experiments with two wells per condition, and the values reported are means ± standard errors.

### Statistics

Statistical comparisons, in the viability test or the invasion assay, were performed using first a generalized linear model and then a Newman-Keuls test. Statistics were conducted assuming equal variance, unpaired samples, and using a 2-tailed distribution. For statistical analysis of sporozoite viability prior to the invasion assay, we used a one-way analysis of variance.

Differences were considered to be statistically significant at a P value<0.05.
